# Parasitic Prevalence in a Suburban School of Famaillá, Tucumán, Argentina

**DOI:** 10.5402/2012/560376

**Published:** 2012-06-26

**Authors:** Julián Dib, Juana Oquilla, Silvia G. Lazarte, Silvia N. Gonzalez

**Affiliations:** ^1^Facultad de Bioquímica, Química y Farmacia, Universidad Nacional de Tucumán, Ayacucho 471, T4000INI San Miguel de Tucumán, Argentina; ^2^CONICET-CCT Tucumán, Chacabuco 145 T4000ILC San Miguel de Tucumán, Argentina

## Abstract

Prevalence of intestinal parasites was investigated in rural primary school children in Famaillá city, Tucumán province, Argentina. Stool specimens from 149 school children were collected. The prevalence rate of intestinal parasite infections was 86.6%. No significant differences were observed in the distribution by age or by sex. *Blastocystis hominis* was the most commonly found protozoan parasite (54.4%), followed by *Entamoeba coli* (35.6%), *Giardia lamblia* (24.8%), and others (16.7%). *Enterobius vermicularis* was the most prevalent intestinal helminth (27.5%), followed by *Ascaris lumbricoides* (20.8%), *Trichuris trichiura* (12.8%), and others (5.4%). Most of the patients had polyparasitism (62.4%), and protozoan infections prevailed over helminthic infections. These results show high rates of parasitism in the school children of Famaillá, which would be associated with socioeconomic factors and poor environmental sanitation conditions in this area.

## 1. Introduction

The World Health Organization states that primary school children population is the most vulnerable group to acquire infection diseases such as parasites while their development is related to the environmental contamination as well as to the quality of consumed food [1].

From an epidemiological, socioeconomical, and ecological point of view, the county populations possess favourable conditions that allowed children to acquire intestinal infections with a greater frequency. Particularly, in Famaillá city, in the province of Tucumán, Argentina, there is little information related with epidemiologic data on children parasitoses. For that reason, the present work deals with the study of parasite-related infections detected in primary school students from the Institution no. 124, in Famaillá. The aim of this work was to assess the prevalence of intestinal parasites in the former population in order to obtain accurate epidemiological data that can be used to program prevention schemes in this particular age group.

## 2. Materials and Methods

For the cohort study, a group of 149 children from kindergarten and primary level of the school no. 124, Famaillá city, Tucumán, was selected and monitored through the summery station. Famaillá, located 30 Km in the south of Tucumán capital district, has 32.000 habitants. The Famaillá district is situated in the central area of the province, limited at the North with the Lules district, at the East with the Leales district, at the South with the Monteros district, and at the West with the Tafí del Valle district.

The personal affiliation (name, sex, and age) of each student was registered. A serial stool specimen was requested from each person, and the anal swab method for *Enterobius vermicularis* eggs was used. Each student was provided with two bottles containing 5% formalin, and an informative-explicative meeting for children, parents, and teachers was conducted prior to the sampling process.

After collecting the stool samples, they were analysed at the lab station by direct smear and through the concentration method described by Melvin and Brooke [2].

The diagnostic elements were visualized with and without staining by lugol or carbol fuchsine by optic microscopy. Formalin-preserved stool samples and the modified Ziehl-Neelsen carbol-fuchsine staining of formalin-ether concentrates were used from the recovery and identification of *Cryptosporidium *spp. oocysts [3].

For *Strongyloides stercoralis* diagnosis the stool samples were subjected to the Koga agar plate method [4].

From the total 149 children selected for this study, 70 were females (47%) and the rest males (53%). Their age ranged from 4 to 14 years.

The studies were adjusted to the conditions of the Universal Declaration of Human Rights (1948), ethical standards instituted by the Nuremberg Code (1947), the Helsinki Declaration (1964), and subsequent amendments regulated by the National Law no. 25,326 related to data protection humans.

## 3. Results and Discussion

The prevalence of intestinal parasites was high (86.6%). No significant differences were found on regard of age and sex of the infected population. 13 endoparasite species were diagnosed but among them the protozoa were more frequently found than the helminths. *Blastocystis hominis* (54.4%), *Entamoeba coli* (35.6%), and *Giardia lamblia* (24.8%) were the most prevalent protozoa. Among helminths, *Enterobius vermicularis* (27.5%) and *Ascaris lumbricoides* (20.8%) were the most frequent (Table 1).

Only 20 children (13.4% of the total population studied) did not present any parasite; in 36 children (24.2%) it was found only one parasite species while 62.4% of the total population was infected by two or more parasite species (Table 2).

It is important to remark that in all cases, the sanitary and social conditions of the studied children habitats were highly inappropriate and most deficient.

It was found that more four-five (86.6%) of the students, aged 4.7 to 14, in the considered school were infected by intestinal parasites. This data is coincident with the data shown by previous works dealing with intestinal parasites in similar populations in Tucumán [5, 6] but the prevalence is higher than the prevalence of these infections in other Argentinean provinces [7, 8]. Compared to other South American countries the prevalence detected in this study can also be considered high as Mercado et al. [9] in Chile and Cabrera et al. [10] in Peru described a 55.8% and 77.8% of intestinal parasite prevalence, respectively.

Among the protozoa, *Blastocystis hominis* was the most prevalent parasite, this concurred with most of the epidemiological studies made in many other countries and it points out that this parasite is an emergent pathogen with the highest prevalence. Milano et al. [8] determined an endoparasite prevalence of 73.5% in 113 infants (between 0 and 14 years) from North East Argentine (NEA) urban area. In that work, *Blastocystis  hominis *(59.0%) and *Enterobius vermicularis* (47.0%) were the more frequent protozoa and helminths, respectively. Menghi et al. [11] studied the prevalence of intestinal parasitoses by protozoans and helminths in an aboriginal community from Salta, Argentine. They found that the more frequent protozoa were *Blastocystis  hominis* (58.9%), *Entamoeba coli *(51.8%), *Giardia lamblia *(27.7%) and *Entamoeba histolytica/E. dispar *(24.1%). In our study, we determined similar prevalence levels of *Blastocystis  hominis* (54.4%) and *Giardia lamblia* (24.8%). A minor incidence of *Entamoeba coli* (35.6%) in school children from Famaillá city was found, and under our work conditions we were unable to identify others species of this protozoan.

Devera et al. [12] have evaluated the prevalence of *Blastocystis hominis* (18.93% ± 5.93%) and its clinical relevance in 162 preschool children living in Bolivar city, Venezuela. In the half of the studied children this protozoan was the only parasite. *Giardia lamblia* was identified with a frequency of 39.13%, and they have concluded that *Blastocystis hominis* was a relatively frequent intestinal parasite among the preschool children evaluated. In addition, the results obtained by Wang et al. [13] showed that *Blastocystis hominis* in humans from Huainan area (703 stool specimens examined) have a very low prevalence (3.70%), which was not related to gender and living circumstances, but they observed statistically significant association between presence of diarrhoea and infection.

Among helminths, the occurrence of *Ascaris lumbricoides* and *Enterobius vermicularis* was important. Our results (27.5%) and those of Milano et al. [8] (47.0%) fully indicated that the prevalence rate of *Enterobius vermicularis* infections was very high compared to other countries. Our present data shows that the prevalence was much higher than that reported in western and southern islands of the Republic of Korea by Park et al. [14] (18.5%), in Aydin, Turkey by Okyay et al. [15] (18.1%); in some rural communities of Ogun State-Nigeria (0.3%) by Agbolade et al. [16], and in Cape Town, South Africa (0.6%) by Adams et al. [17]. The risk of *E. vermicularis* infection among school children can increase by inadequate personal hygiene, the biting nails habit, and not washing hands before meals.

Surprisingly, only one case of hookworm was detected, although historically Famaillá was considered an important area of hookworm prevalence (9.2%). In our work, there was found a lower frequency than in previous works [11, 18], where hookworms (58.0%), *Hymenolepis nana* (31.2%), and *Strongyloides stercoralis* (24.1%) were reported as the most frequent helminths. Anantaphruti et al. [19] detected of 1.8% *Strongyloides stercoralis* infection, in school children, while in this study we found this helminth with a frequency of 2.7%. In addition, Taranto et al. [20] studying the clinical and parasitological status of a Wichi Aboriginal community living in the suburbs of Tartagal, northern Salta, Argentina, determined that the most frequent helminths were *Strongyloides stercoralis* (50.5%). Special techniques [4, 21] are required for detection of *Strongyloides* infection and, for this reason, in past researches, the fecal levels have often been undervalued or ignored. According to Pawlowski [22] all regions cited in this paragraph might be considered endemic areas for *Strongyloides stercoralis* infection because the prevalence rate was determined between 1 and 5%.

Our results indicated that the overall prevalence rate of pinworm infections was rather low and the multiple infections were outstanding because of high frequency (62.4%).

It has been showed before that the main source of infection for protozoa is the ingested water. The high prevalence of this kind of parasites suggests that further studies must be done about the water quality in this community to accurate determination of the infection source of these parasites. This study showed intestinal parasitosis as critical problem of public health in children from areas where the lack of adequate sanitation conditions and unsuitable supply of water today coexist. Intestinal parasitosis is a social problem with important consequences as poor school performance and impaired quality of life. This is one of few studies for determining parasitic prevalence in primary school children of North West Argentine (NOA) region. Because of this, it is very important and we propose that regular screening and treatment programs should be started. In addition, health education on personal hygiene is required and the uneducated women should be aided with specific programs. A multispectral government action is needed.

## Figures and Tables

**Table 1:** Prevalence of intestinal parasites in school children of Famaillá city, Tucumán province, Argentina.

Parasites	Positive cases	Frequency (%)^∗^
Protozoa		
*Giardia lamblia*	37	24.8
*Blastocystis hominis*	81	54.4
*Endolimax nana*	20	13.4
*Entamoeba coli*	53	35.6
*Iodamoeba bütschlii*	2	1.3
*Chilomastix mesnili*	3	2.0

Helminths		
*Enterobius vermicularis*	41	27.5
*Strongyloides stercoralis*	4	2.7
*Ascaris lumbricoides*	31	20.8
*Hymenolepis nana*	3	2.0
*Trichuris trichiura*	19	12.8
Hookworm	1	0.7

^∗^ *n* = 149.

**Table 2:** The rate of mixed parasite infections in school children of Famaillá city, Tucumán province, Argentina.

Species	Positive cases	Frequency (%)^∗^
1	36	24.2
2	47	31.5
3	26	17.4
≥**4**	20	13.4

^∗^ *n* = 149.

## References

[1] World Health Organization

[2] Melvin DM, Brooke MM (1982). *Laboratory Procedures for the Diagnosis of Intestinal Parasites*.

[3] Garcia LS, Bruckner DA, Brewer TC, Shimizu RY (1983). Techniques for the recovery and identification of cryptosporidium oocysts from stool specimens. *Journal of Clinical Microbiology*.

[4] Koga K, Kasuya S, Khamboonruang C (1991). A modified agar plate method for detection of *Strongyloides stercoralis*. *American Journal of Tropical Medicine and Hygiene*.

[5] Lopez C, Cabrera M, Dib JR (2004). Prevalence and infection due to intestinal parasite in children who live in the bank of Canal Norte, S. M. de Tucumán, Argentina. *Biocell*.

[6] Silvia GL, Christian A, Juana O, Silvia NG (2006). Alarming levels of infection by enteric parasites in children of Burruyacu, Province of Tucumán, Argentina. *International Journal of Tropical Medicine*.

[7] Bontti S (1999). Enteroparasitoses en el Departamento de Lavalle, Mendoza. *Medicina*.

[8] Milano AMF, Oscherov EB, Palladino AC, Bar AR (2007). Children enteroparasitosis in North East Argentine urban area. *Medicina*.

[9] Mercado R, Castillo D, Muños V (2003). Infecciones por protozoos y helmintos intestinales en pre-escolare y escolares de la Comuna de Colina, Santiago, Chile. *Parasitología Latinoamericana*.

[10] Cabrera M, Verástegui M, Cabrera R (2005). Prevalencia de enteroparásitos en una comunidad altoandina de la Provincia de Victor Fajardo, Ayacucho, Perú. *Revista de Gastroenterología del Perú*.

[11] Menghi CI, Iuvaro FR, Dellacasa MA, Gatta CL (2007). Investigación de parásitos intestinales en una comunidad aborigen de la provincia de Salta. *Medicina*.

[12] Devera RA, Velásquez VJ, Vasquez MJ (1998). Blastocystosis en pre-escolares de Ciudad Bolívar, Venezuela. *Cadernos de Saúde Pública*.

[13] Wang KX, Li CP, Wang J, Cui YB (2002). Epidemiological survey of Blastocystis hominis in Huainan City, Anhui Province, China. *World Journal of Gastroenterology*.

[14] Park JH, Han ET, Kim WH (2005). A survey of *Enterobius vermicularis* infection among children on Western and Southern coastal islands of the Republic of Korea. *The Korean Journal of Parasitology*.

[15] Okyay P, Ertug S, Gultekin B, Onen O, Beser E (2004). Intestinal parasites prevalence and related factors in school children, a Western city sample-Turkey. *BMC Public Health*.

[16] Agbolade OM, Agu NC, Adesanya OO (2007). Intestinal helminthiases and schistosomiasis among school children in an urban center and some rural communities in Southwest Nigeria. *The Korean Journal of Parasitology*.

[17] Adams VJ, Markus MB, Adams JFA (2005). Paradoxical helminthiasis and giardiasis in Cape Town, South Africa: epidemiology and control. *African Health Sciences*.

[18] Valperga SM, Pérez L, Nava CA (1979). Helmintiasis intestinales en habitantes del ex departamento Famaillá (Provincia de Tucumán). *Revista de la Facultad de Medicina Tucumán*.

[19] Anantaphruti MT, Nuamtanong S, Muennoo C, Sanguankiat S, Pubampen S (2000). Strongyloides stercoralis infection and chronological changes of other soil-transmitted helminthiases in an endemic area of southern Thailand. *Southeast Asian Journal of Tropical Medicine and Public Health*.

[20] Taranto NJ, Cajal SP, De Marzi MC (2003). Clinical status and parasitic infection in a Wichí Aboriginal community in Salta, Argentina. *Transactions of the Royal Society of Tropical Medicine and Hygiene*.

[21] Sato Y, Kobayashi J, Toma H, Shiroma Y (1995). Efficacy of stool examination for detection of *Strongyloides* infection. *American Journal of Tropical Medicine and Hygiene*.

[22] Pawlowski ZS, Grove DI (1989). Epidemiology, prevention and control. *Strongyloidiasis: A Major Roundworm Infection of Man*.

